# Ibudilast‐Mediated Suppression of Neuronal TLR4 in the Prefrontal Cortex Mitigates Methamphetamine‐Induced Neuroinflammation and Addictive Behaviours

**DOI:** 10.1111/adb.70033

**Published:** 2025-04-16

**Authors:** Fangmin Wang, Huizhen Liu, Yuting Ke, Xiaolei Huang, Shanshan Chen, Dingding Zhuang, Yiying Zhou, Manqing Wu, Yuting Wang, Miaojun Lai, Huifen Liu, Wenhua Zhou

**Affiliations:** ^1^ Zhejiang Provincial Key Lab of Addiction Research The Affiliated Kangning Hospital of Ningbo University Ningbo People's Republic of China; ^2^ Department of Psychiatry The Affiliated Kangning Hospital of Ningbo University People's Republic of China; ^3^ Department of Obstetrics The Affiliated Lihuili Hospital of Ningbo University Ningbo P. R. China; ^4^ Shanghai Mental Health Center Shanghai People's Republic of China; ^5^ Department of Biological Engineering Massachusetts Institute of Technology Cambridge Massachusetts USA

**Keywords:** addiction, drug use disorders, methamphetamine, microglia, psychostimulant, toll‐like receptor

## Abstract

Methamphetamine (METH) use leads to addiction, neurotoxicity, and neuroinflammation. Ibudilast, a toll‐like receptor 4 (TLR4) inhibitor, has been shown to reduce METH‐induced neuroinflammation and self‐administration, but its specific role in neuronal TLR4 signalling and associated behavioural outcomes remains poorly understood. This study examined Ibudilast's effects on METH reward, drug‐seeking behaviour, and TLR4 signalling in a rat self‐administration model. Ibudilast was found to dose‐dependently reduce METH intake and motivation for the drug, as evidenced by a downward shift in the dose–response curve and a decrease in breakpoint. Additionally, Ibudilast suppressed both cue‐ and METH priming‐induced drug‐seeking behaviours. Western blot analysis revealed elevated TLR4, p‐NF‐κB and IL‐6 in the prefrontal cortex after 14 days of METH self‐administration. These increases were significantly attenuated by Ibudilast treatment. Furthermore, local administration of Ibudilast in the prefrontal cortex led to a reduction in METH intake and motivation, as well as decreased TLR4 expression in this brain region. Immunofluorescence staining was revealed that TLR4 was expressed predominantly in neurons and microglia, with METH‐induced upregulation of neuronal TLR4 being linked to apoptosis. Ibudilast restored normal spatial interactions between neurons and microglia, thereby mitigating neuroinflammation and neuronal damage. Furthermore, local injection of Ibudilast in the prefrontal cortex led to a reduction in METH intake and motivation, as well as decreased expression of TLR4 in the brain region. These findings underscore the critical role of neuronal TLR4 in METH addiction and highlight Ibudilast's therapeutic potential in addressing METH‐related neuroinflammation and behavioural dysregulation.

## Graphical abstract

This graphical abstract illustrates the neuronal TLR4‐mediated neuroinflammatory cascade in the prefrontal cortex of methamphetamine‐addicted rats. Using a traffic light metaphor, it demonstrates how: (1) TLR4 overexpression (red light) triggers microglial recruitment; (2) activated microglia (amoeboid shape) amplify neuroinflammation; (3) neuronal apoptosis and phagocytosis underscore the critical role of TLR4 dysregulation in methamphetamine addiction.

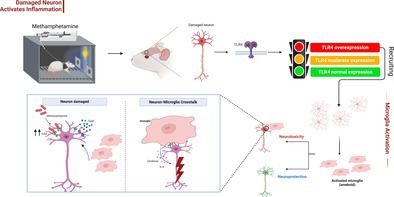

## 1 Introduction

Methamphetamine (METH) is a highly addictive and neurotoxic psychostimulant that presents significant public health challenges due to its widespread abuse and the severe neurological and cognitive deficits it induces. Chronic METH use not only leads to addiction and substantial socioeconomic costs but also contributes to neuroinflammation, oxidative stress and neuronal apoptosis, all of which are key components of its neurotoxicity profile [1]. Toll‐like receptor 4 (TLR4), a pattern recognition receptor, plays a central role in mediating inflammatory responses to a range of endogenous and exogenous toxins, including drugs of abuse [2]. Within the central nervous system, TLR4 is chiefly expressed in non‐neuronal cells, such as microglia and astrocytes, where it plays a key role in immune processes, particularly in the secretion of pro‐inflammatory cytokines and microglial phagocytosis [3]. However, the expression of TLR4 in neurons and its involvement in neuroinflammatory pathways remain a subject of ongoing debate [4].

Historically, it was believed that neurons do not significantly express TLR4; however, emerging evidence suggests that neurons may express TLR4 under certain pathological conditions or in specific brain regions [5, 6]. For example, studies have shown that TLR4 is expressed in neurons during stress conditions such as cerebral ischemia, neuroinflammation and certain neurodegenerative diseases like Alzheimer's disease [7, 8]. A growing body of research indicates that psychostimulants, such as cocaine and METH, activate and modulate neuroimmune responses [9]. Chronic exposure to METH activates TLR4 in glial cells, leading to the release of pro‐inflammatory substances [10]. Additionally, TLR4 activation could potentially induce apoptosis or excitotoxicity, which damages neurons [11].

Although sporadic reports indicate that the TLR4 inhibitor Ibudilast inhibits METH‐induced motor sensitization and reinforcing effects [12], the mechanisms by which Ibudilast modulates neuronal TLR4 pathways in METH addiction have yet to be fully elucidated. The prefrontal cortex (PFC) is a critical component of the dopaminergic circuit and plays a crucial role in regulating cognitive behaviour, emotional function, decision‐making, consciousness and language processing [13]. Moreover, the PFC is considered a central region of the brain's reward system, responsible for mediating addiction to both natural rewards (such as food and sleep) and pharmacological stimuli (such as opioids) [14]. Dysfunction in the PFC can lead to impulsive behaviours, obsessive‐compulsive disorders and attention deficits, all of which are associated with the loss of vehicle over drug‐seeking behaviours [15, 16, 17].

The IL region of the PFC has been shown to be involved in regulating emotional responses and executive functions, which are disrupted in individuals with substance use disorder [18]. This region is considered a key mediator in the brain's reward system and is particularly important in the regulation of drug‐seeking and relapse behaviours, making it a crucial target in addiction research [19]. Emerging evidence suggests that the activation of TLR4 in the IL region could modulate neuroinflammatory responses that contribute to the reinforcement of addictive behaviour [20]. Therefore, understanding the role of TLR4 signalling in neurons of the IL region may provide important insights into the neurobiological mechanisms underlying METH addiction.

Based on previous research, we hypothesize that TLR4 in neurons may play an important role in mediating the rewarding effects of METH [21, 22]. Our objective is to inhibit TLR4 using Ibudilast and explore whether this inhibition can reduce METH reward behaviour by decreasing the nuclear translocation of NF‐κB downstream of neuronal activation [23]. To achieve this, we will first examine the effects of Ibudilast on METH self‐administration, reward motivation, relapse behaviours, and natural reward behaviours, and correlate these effects with inflammatory markers [24]. Additionally, we will link these effects to the interactions between neurons and glial cells and investigate neuronal apoptosis [25].

## 2 Materials and Methods

### 2.1 Animals

Male Sprague–Dawley (SD) rats, weighing between 250 and 280 g, were sourced from the Zhejiang Experimental Animal Center (License No: SCXK [Zhejiang] 2017–0033). Upon their arrival at the Animal Experimental Center of Ningbo Kangning Hospital, the rats were kept in a controlled clean environment with a 12 h light/dark cycle (lights off at 8:00 PM), a stable temperature range of 22°C–24°C, 50%–70% humidity, and adequate ventilation. The animals had unrestricted access to distilled water and were given sterilized feed (processed through high‐temperature, high‐pressure sterilization). Bedding and housing areas were routinely disinfected to maintain cleanliness. This study was conducted in strict accordance with the Regulations for the Administration of Experimental Animals in the People's Republic of China and received approval from the Animal Ethics Committee of Ningbo City, Zhejiang Province.

### 2.2 Chemical Reagents

METH was provided by the Beijing Drug Enforcement Administration and dissolved in sterile saline (Shuanghe, China). It was diluted to concentrations of 0.05 and 0.5 mg/kg for self‐administration training and drug priming experiments, respectively. Prior to use, the METH solution was filtered through a Millex‐GP filter, 0.22 μm, PES 33 mm (Merck, Germany) for sterilization. Ibudilast (3‐isobutyryl‐2‐isopropylpyrazolo[1,5‐a]pyridine) purchased from MCE (Shanghai, China) was dissolved in 10% Dimethyl Sulfoxide (DMSO), 40% Polyethylene Glycol 300 (PEG300), 5% Tween‐80 and 45% saline and diluted to concentrations of 3 and 10 mg/kg for intraperitoneal (i.p.) injection.

### 2.3 Jugular Catheterization Surgery

Rats weighing 280‐300 g were acclimated to a 12 h light/dark cycle in a specific pathogen‐free (SPF) animal facility for 7 days before undergoing jugular vein catheterization surgery. The rats were anaesthetised by intramuscular injection of Zoletil (0.1 mg/kg, i.p.). To prevent asphyxiation, 0.3 mg/kg of atropine sulphate was administered 15 min before anaesthesia. The jugular vein was located and carefully dissected in the jugular vein triangle area. A 45° angle incision was made along the transverse plane of the jugular vein, and the catheter (a 4 cm silicone tube and a 10 cm polyethylene tube, PE20) was inserted using a guide needle. Once secured, the catheter was tunnelled subcutaneously along the rat's back through a skin puncture and covered with a custom‐made vest. To prevent infection at the catheter site, 400 000 units of penicillin solution (0.3 mL) were injected. Following surgery, the rats were housed in a temperature‐controlled environment for 3–5 days, with unrestricted access to food and water. Daily injections of a mixture consisting of 1:100 heparin and 400 000 units of sodium penicillin (0.3 mL) were given through the catheter to prevent infection and ensure catheter patency for successful drug administration.

### 2.4 METH Self‐Administration Training

Following previously published protocols (13), rats underwent METH self‐administration training using a fixed ratio (FR1) reinforcement schedule, where METH (0.05 mg/kg per infusion) injections were triggered by a single active nose‐poke. Upon the start of the session, the light in the cage illuminated, accompanied by the sound of the drug pump whenever the rat made an active nose poke, resulting in the delivery of a METH injection. After the injection, a 20‐s timeout period ensued, during which any further active nose‐pokes were recorded but did not lead to additional METH infusions. Once the timeout period ended, the cage light turned off, and the next cycle began. Throughout the training period, rats were deprived of food and water and trained daily for 4 h (from 10:00 AM to 2:00 PM), with free access to 20 g of food and water during non‐training hours. Active nose‐pokes and injections were recorded until a stable high level of active nose‐pokes (50 ± 10) and a reduction in inactive nose‐pokes (10 ± 5) were achieved, indicating the successful establishment of the self‐administration model. The typical training period lasted for 14 days.

#### 2.4.1 The Effects of Different Doses of Ibudilast on METH Self‐Administration

Rats that successfully developed the METH self‐administration model were randomly assigned to three groups (*N* = 6) for treatment with different doses of Ibudilast via intraperitoneal (i.p.) injection. The groups included a Vehicle group receiving 10% DMSO, 40% Polyethylene Glycol 300 (PEG300), 5% Tween‐80 and 45% saline injections, a low‐dose group receiving 3 mg/kg Ibudilast, and a high‐dose group receiving 10 mg/kg Ibudilast. Treatment consisted of daily ip injections for three consecutive days, with each group receiving either saline, 3 mg/kg Ibudilast, or 10 mg/kg Ibudilast. Injections were administered 30 min before the METH self‐administration session. After the injection, the rats were returned to their training cages for a 4‐h METH self‐administration session. During this period, the number of active and inactive nose‐pokes, as well as the number of injections, was recorded daily to assess the effects of Ibudilast at different doses on METH self‐administration behaviour.

#### 2.4.2 Motivation Assessment During METH Self‐Administration

Once a stable METH self‐administration model is established, motivation can be assessed using the progressive ratio (PR) procedure. Unlike the fixed ratio (FR) procedure, where a single active nose‐poke triggers one METH infusion, the PR procedure requires an increasing number of active nose‐pokes for each subsequent METH infusion. This method helps assess the rat's motivation and craving by progressively increasing the difficulty of obtaining the drug.

The response ratio is calculated with the following formula: Response ratio = [5e^(number of injections × 0.2)] − 5. For instance, the first infusion requires 1 active nose‐poke, the second requires 2, the third requires 4, and so on, following this sequence: 1, 2, 4, 5, 8, 11, 15, 20, 24, 32, 40, 49, 62, 77, 95, 116, 145, 178, 209, 268, 329, 402, 492, 603, etc. The breakpoint refers to the total number of infusions obtained before the rat discontinues self‐administration, while the final ratio is the number of active nose‐pokes completed in the last session.

Typically, the PR procedure is conducted for 6 h, but in this experiment, it lasted for 4 h. If the rat does not respond within 1 h (i.e., no drug infusion), the procedure automatically terminates.

#### 2.4.3 Effects of Different Doses of Ibudilast on METH Reward Motivation

On the fourth day of Ibudilast treatment, the three groups of rats underwent motivation testing using the PR procedure. Prior to the METH training session, the three groups received intraperitoneally 10% DMSC, 40% Polyethylene Glycol 300 (PEG300), 5% Tween‐80, and 45% saline, 3 or 10 mg/kg Ibudilast 30 min before the session. After 30 min, the rats were returned to their self‐administration training cages to undergo a 4‐h METH self‐administration session using the PR procedure. During this period, data on the number of active nose‐pokes, inactive nose‐pokes, and injections were collected to evaluate the effects of different doses of Ibudilast on METH reward motivation.

#### 2.4.4 Effects of Ibudilast on Dose‐Effect Curve of METH Self‐Administration

Under the FR1 schedule, rats underwent METH self‐administration training. Once a METH addiction model was successfully established, self‐administration behavioural experiments continued using multiple doses of METH (0, 0.003125, 0.00625, 0.0125, 0.025, 0.05, 0.1, 0.2 mg/kg). Initially, an extinction trial was conducted with 0 mg/kg METH injections, lasting 25 min. Following this, six dosage trials were performed sequentially in ascending order of METH doses, with each trial lasting 25 min and separated by a 5‐min intertrial interval. METH doses were determined by adjusting the injection volume of the METH solution.

Once stable METH self‐administration behaviour was achieved, pretreatment tests with Ibudilast were initiated. Stability of behaviour was defined as: (1) rats consuming at least 5 mg/kg of METH per session, with the total number of injections varying by no more than 20% across two consecutive sessions; and (2) the dose of METH maintaining the maximum response rate changing by no more than half a logarithmic unit across two consecutive sessions, consistent with previous studies (14, 15).

Thirty minutes before each session, rats received pretreatment with Ibudilast (0, 3, or 10 mg/kg, i.p.) or Vehicle. The experimental sequence was randomized to balance the testing order of different Ibudilast doses. After each Vehicle or Ibudilast pretreatment, rats resumed the progressive‐dose METH self‐administration protocol until their behaviour stabilized, after which subsequent Ibudilast or Vehicle doses were tested under the same conditions.

### 2.5 Withdrawal, Extinction, and Reinstatement of METH Addiction

After successful establishment of the model, the rats were returned to their original individual cages and no more METH was administered. They entered a 14‐day abstinence period during which they had free access to food and water, and the light–dark cycle and environmental conditions remained constant. This period was referred to as the abstinence phase. Upon completion of the abstinence phase the next steps were initiated.

Following the extinction phase, METH reinstatement was conducted, which involved cue‐induced reinstatement and drug‐induced reinstatement. Cue‐induced reinstatement consisted of context cue (CC) and conditional cue (CS) inductions. The CC referred to the training cage in which the rats received self‐administration training, without cage lights or pump sounds and the light for valid nose pokes was turned off. When the rats made a valid nose poke in the CC condition, the METH pump did not function, and there were no associated cues. The computer continued to record the number of valid and invalid nose pokes, similar to the extinction experiment procedure. The CS represented the conditions that were identical to the self‐administration training, including the training cage and associated lights and sounds. On the 15th day of abstinence, a 2‐h reinstatement test under CC induction was conducted. During the 2‐h CC session, the cues associated with the training cage were turned off, and the numbers of valid and invalid nose pokes were recorded for behavioural analysis.

The CS induction replicated the self‐administration training conditions. When the 2‐h CS session was initiated, cage lights, nose poke lights and pump sounds within the training cage were activated. When the rats made a valid nose poke, the subsequent context was consistent with the self‐administration training, and a single CS cue, including cage lights and pump sounds, was presented, without METH administration. The computer continued to record the number of valid and invalid nose pokes for behavioural analysis. Reinstatement of METH‐induced behaviour included both pure CC‐induced reinstatement and CS‐induced reinstatement.

Throughout these procedures, the number of active and inactive nose pokes was recorded in the computer programme to facilitate the analysis of rat behaviour.

#### 2.5.1 Effect of Ibudilast on Extinction of Drug Seeking in Training Context

Rats that completed the 2‐h context cue (2hCC) withdrawal were randomly assigned to two groups (*N* = 6), namely, the Vehicle (i.p.) administration group and the 3 mg/kg Ibudilast i.p. administration group. For three consecutive days prior to the 2 h CC test, the two groups of rats received i.p. injections of Vehicle or 3 mg/kg Ibudilast. On the third day, after i.p. injections of Vehicle or 3 mg/kg Ibudilast, the rats were placed in their respective self‐administration training cages and the 2 h CC test programme was initiated. During this period, data on valid nose pokes were collected to evaluate the effect of 3 mg/kg Ibudilast on environment cue‐induced METH reinstatement behaviour.

#### 2.5.2 Effect of Ibudilast on Reinstatement Induced by Conditioned Cues

Rats that completed the 2‐h conditioned cue (2hCS) withdrawal were randomly assigned to two groups (*N* = 6), namely, the Vehicle (i.p.) administration group and the 3 mg/kg Ibudilast i.p. administration group. For three consecutive days prior to the 2 h CS test, the two groups of rats received i.p. injections of Vehicle or 3 mg/kg Ibudilast. On the third day, after i.p. injections of Vehicle or 3 mg/kg Ibudilast, the rats were placed in their respective self‐administration training cages and the 2 h CS test programme was initiated. During this period, data on valid nose pokes were collected to evaluate the effect of 3 mg/kg Ibudilast on conditioned cue‐induced METH reinstatement behaviour.

#### 2.5.3 Effect of Ibudilast on Reinstatement Induced by METH Priming

Rats that completed the 2 h CS withdrawal following drug priming were randomly assigned to four groups (*N* = 6), namely, the Vehicle i.p. administration group, 0.5 mg/kg METH i.p. administration priming group, 3 mg/kg Ibudilast i.p. administration group and the combined 0.5 mg/kg METH i.p. administration priming group with 3 mg/kg Ibudilast i.p. administration. Forty‐five minutes prior to the 2 h CS test, the 3 mg/kg Ibudilast i.p. administration group and the combined 0.5 mg/kg METH i.p. administration priming group with 3 mg/kg Ibudilast i.p. administration received their respective drug treatments. Thirty minutes later, the 0.5 mg/kg METH i.p. administration priming group and the combined 0.5 mg/kg METH i.p. administration priming group with 3 mg/kg Ibudilast i.p. administration was administered METH via i.p. injections. Fifteen minutes later, the rats were placed in their respective self‐administration training cages and the 2 h CS test programme was initiated. During this period, data on valid nose pokes were collected to evaluate the effect of 3 mg/kg Ibudilast on drug cue‐induced METH reinstatement behaviour.

### 2.6 Sucrose Reinforcement Training (Food Reward)

One experimental session was conducted per day. The protocol specified that during each session, rats would receive one sucrose reinforcement for successfully completing a nose poke. The session would automatically terminate when rats completed 100 reinforcements or when the training duration reached 1 h. Starting from FR1 (one nose poke resulting in one sucrose pellet reinforcement), if all rats completed 100 sucrose reinforcements in two consecutive sessions, the reinforcement ratio was increased. The training then progressed to FR2, FR3, FR5 and finally FR10 for sucrose reinforcement.

#### 2.6.1 Effect of Different Doses of Ibudilast on Food Reward

Rats trained with FR10 for food reinforcement were randomly divided into three groups (*N* = 6): the Vehicle (i.p.) injection Vehicle group, the low‐dose 3 mg/kg Ibudilast i.p. treatment group and the high‐dose 10 mg/kg Ibudilast i.p. treatment group. The treatment was administered continuously for 3 days. During these 3 days, the three groups of rats received i.p. injections of Vehicle, 3 mg/kg, or 10 mg/kg Ibudilast, respectively. The i.p. injections were administered 30 min before each food training session, and 30 min later, the rats were returned to their self‐administration training cages for food reward training. During this period, data were collected on the number of valid nose pokes, invalid nose pokes and the number of sucrose pellets consumed to evaluate the effect of different doses of Ibudilast on food reward.

### 2.7 Western Blot

Proteins were extracted from PFC tissue by adding pre‐thawed and completely thawed SDS lysate, which was used in the ratio of 20 mg:1 mL, along with the protease inhibitor PMSF in the ratio of 1:100 and the phosphatase inhibitor. After electrophoresis, membranes were transferred, primary antibody (TLR4, 1:200–1:500, PA523124, Invitrogen； NF‐κB p65, 1:1000, #8242, Cell Signalling, Phospho‐NF‐κB p65) (1:500; MA5–15160; Invitrogen, USA) IL‐6 (1:1000; ab9324; Abcam, UK) β‐actin (1:5000; AC026; ABclonal, China) was added and secondary antibody (Goat anti‐Rabbit HRP‐linked antibody, 1:5000, #7074, Cell Signalling) was added. Scanning was performed using a chemiluminescence scanner, and the images were scanned using Image J processing software, based on the grayscale values of the bands of the target and internal reference proteins, in order to correct errors, the ratio of the grayscale value of the target protein to the grayscale value of the internal reference protein, β‐actin, needed to be calculated.

### 2.8 Immunofluorescence

The paraffin slices were deparaffinized and rehydrated using 1 × citrate buffer solution (pH = 6.0) for antigen retrieval. The slides were soaked in a 0.5% Triton X‐100 solution for cell permeabilization then incubated in a 3% hydrogen peroxide solution to remove endogenous peroxidase. After blockade with 3% BSA for 1 h at room temperature, the slides were incubated with the primary antibody (IBA1, Ab283319, Abcam; IBA1, Ab178846, Abcam; NeuN, ABN78, Sigma‐Aldrich; GFAP, PA1–10004, Thermo Fisher； TLR4, 1:200–1:500, PA523124) was incubated overnight at 4°C. Subsequently, the secondary antibody (Goat anti‐Mouse IgG (H + L) Highly Cross‐Adsorbed Secondary Antibody, Alexa Fluor Plus 488, A11001, Thermo fisher; Goat anti‐rabbit IgG (H + L) Highly Cross‐Adsorbed Secondary Antibody, Alexa Fluor Plus 594, A11012, Thermo fisher) was added and incubated for 1 h at 37°C. Finally, the slides were observed and imaged using an Olympus upright microscope BX41.

#### 2.8.1 Effects of Microinjection of Ibudilast in the Infralimbic Cortex on METH Reinforcement

Adult SD rats, after being anaesthetised with Zoletil (0.1 mg/kg, i.p.), were carefully positioned in a stereotaxic apparatus (Stoelting 51 950) manufactured in the USA for precise intracranial surgical procedures. During the surgery, a 24‐gauge thin‐walled stainless steel cannula (provided by RWD Life Science Co. China) was used as a guide cannula and was accurately bilaterally implanted 0.5 mm above the IL region in the rats' brains (specific coordinates: anterior +3.0 mm, mediolateral ± 0.8 mm, dorsoventral −5.6 mm). To ensure the stability of the cannula, stainless steel screws and dental cement were used for fixation. Following this, the rats were given at least a 7‐day recovery period. After the recovery period, the rats were randomly divided into three groups, with six rats in each group: the sham group, the group receiving Vehicle per side and the group receiving 5 μg/μL of Ibudilast per side. Thirty minutes before the experiment, Ibudilast was injected into the rats' brains at a constant rate of 0.1 μL/min. Upon completion of the injection, a 5‐min waiting period elapsed before the needle was carefully and slowly removed.

### 2.9 Statistical Methods

The protein grayscale values were analysed using Image‐Lab software. Statistical analyses of the experimental data were performed using SPSS 18.0 software, and the statistical graphs were plotted using GraphPad Prism 5.0 software. The experimental results are presented as mean ± standard deviation. Multiple group comparisons were conducted using one‐way analysis of variance (one‐way ANOVA) or two‐way analysis of variance (two‐way ANOVA). The differences between two groups were analysed using *t*‐test. A significance level of *p* < 0.05 was considered statistically significant.

## 3 Results

### 3.1 Ibudilast Dose‐Dependently Decreased METH Intake and Motivation for METH and Produced a Downward Shift in the METH Dose–Response Curve

**FIGURE 1:**
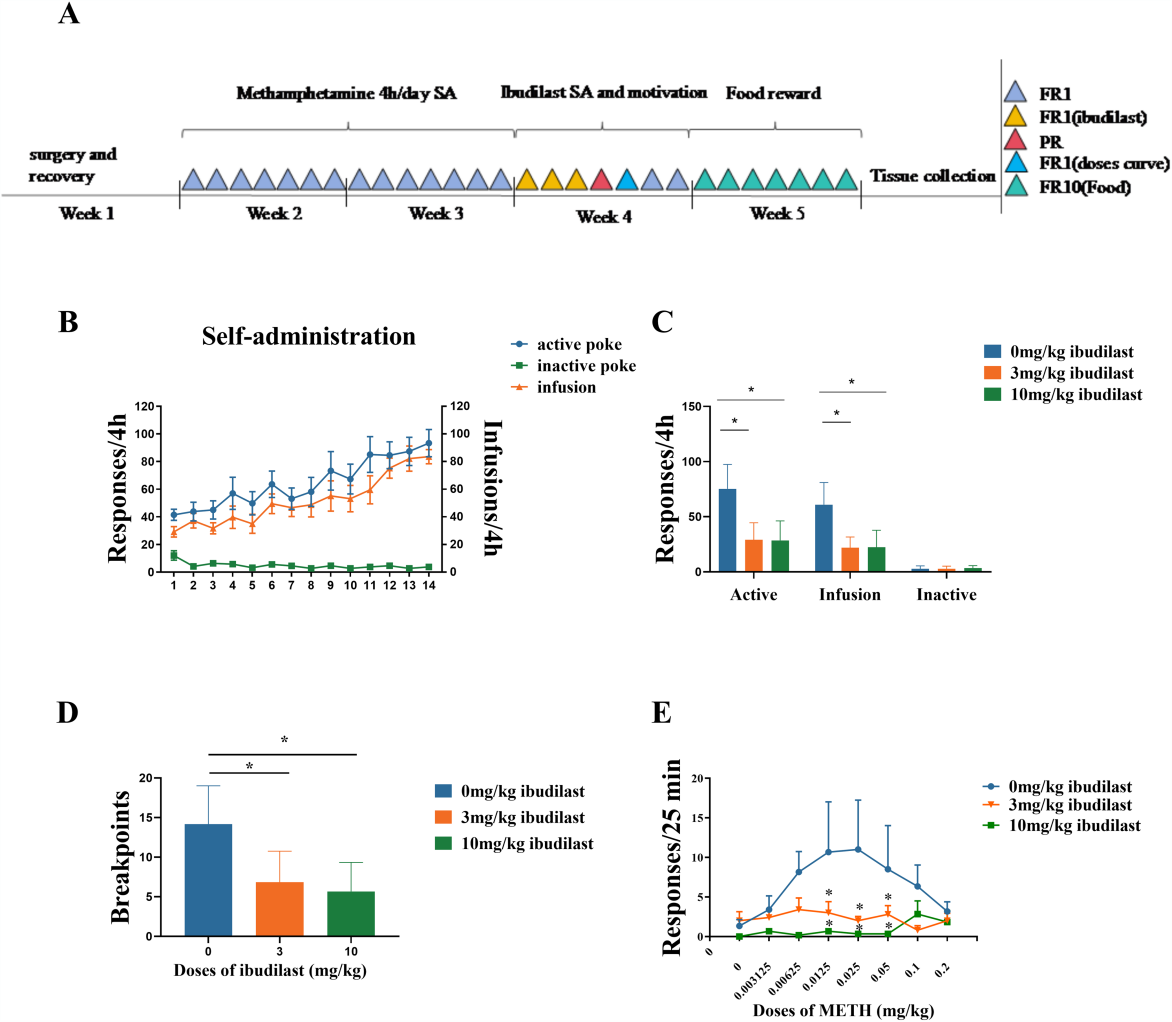
Effects of Ibudilast on METH intake, motivation and dose–response curve in rats. **(A)** Schematic representation of the experimental design and procedure. **(B)** Establishment of the methamphetamine self‐administration model in rats. **(C)** Responses during the 4‐h session, including active pokes, inactive pokes, and infusion counts for rats treated with 3 and 10 mg/kg Ibudilast. **(D)** Breakpoints determined via the PR procedure. **(E)** Dose–response curve for methamphetamine in rats treated with 3 and 10 mg/kg Ibudilast. Data are presented as mean ± SD (*n* = 6). *p < 0.05, 3 or 10 mg/kg Ibudilast vs. Vehicle.

The experimental design of this study is shown in Figure 1A. After completing the cervical cannulation surgery, 280 g SD rats (*n* = 6 per group) underwent self‐administration training under the FR1 schedule for 10 days, with a daily 4‐h training session. Following the training, the rats were administered Ibudilast for 3 days, after which motivation testing and dose–response experiments were conducted. After recovery, the rats underwent natural reward testing.

We first aimed to investigate the effects of Ibudilast on METH self‐administration in rats under the FR1 schedule. Rats rapidly acquired the discrimination between active and inactive nose pokes over a 14‐day training period and achieved a stable METH self‐administration model (Figure 1B). Thirty minutes prior to the self‐administration tests on days 11–13, rats were administered with Ibudilast (3, or 10 mg/kg, i.p.) or Vehicle (i.p.). The doses of Ibudilast and the pretreatment time were based on previous studies1. As shown in Figure 1C, Ibudilast pretreatment exerted a significant dose‐dependent inhibitory effect on active nose‐pokes (F _(2, 15)_ = 11.25, *p* = 0.002) and METH infusions (F _(2, 15)_ = 13.63, *p* = 0.001) but had no significant impact on inactive nose‐pokes (F _(2, 15)_ = 0.195, *p* = 0.825). Post‐hoc Tukey's tests revealed that compared to Vehicle pretreatment, the 3 mg/kg and 10 mg/kg doses of Ibudilast significantly reduced active nose pokes (*p* = 0.007; *p* = 0.021, respectively, Figure 1C) and METH infusions (*p* = 0.014, *p* = 0.007, Figure 1C) on the third day. Next, we examined whether Ibudilast could affect the motivation for self‐administering METH by conducting experiments under the PR schedule. In this experiment, rats had to achieve progressively increasing numbers of active nose pokes to receive the corresponding reward (METH infusion) until reaching a breakpoint level. On day 14, the effects of Ibudilast (3 or 10 mg/kg, i.p.) or a Vehicle on the METH breakpoint were examined. ANOVA rank test revealed that Ibudilast dose‐dependently decreased the METH breakpoint (F _(2, 15)_ = 7.321, *p* = 0.006; Figure 1D). Post‐hoc Tukey's tests showed that compared to Vehicle pretreatment, Ibudilast pretreatment significantly reduced the breakpoint at the 3 (*p* = 0.016) and 10 mg/kg doses (*p* = 0.006). There was no significant difference in the breakpoint between the 3 and 10 mg/kg doses of Ibudilast (*p* = 0.880).

To test the hypothesis that Ibudilast attenuates the reinforcing effects of METH, we examined the effects of Ibudilast on multi‐dose METH self‐administration. Once stable self‐administration behaviour was achieved, rats were switched to multi‐dose METH self‐administration maintained by a series of different doses in a single session (see Section 2). The dose–response curve for METH exhibited an inverted U‐shape, with the maximum infusion occurring at 0.025 mg/kg/infusion (Figure 1E). Prior to the multi‐dose METH self‐administration, Ibudilast (3 or 10 mg/kg, i.p.) or Vehicle injections were administered. As shown in Figure 1E, we found that under the FR1 schedule, Ibudilast caused a dose‐dependent downward shift in the METH dose–response function. ANOVA revealed that 3 mg/kg of Ibudilast significantly suppressed the number of injection responses for METH at concentrations of 0.0125 (*p* = 0.032), 0.025 (*p* = 0.0384) and 0.05 mg/kg (*p* = 0.041). Additionally, 10 mg/kg of Ibudilast significantly inhibited the number of injection responses for METH at concentrations of 0.0125 (*p* = 0.0373), 0.025 (*p* = 0.0395) and 0.05 mg/kg (*p* = 0.0401). These findings further support the notion that Ibudilast could reduce the rewarding and dependency potential of METH.

### 3.2 Effects of Ibudilast on Drug‐Seeking Induced by Cues or METH Priming and Oral Sucrose Self‐Administration

**FIGURE 2:**
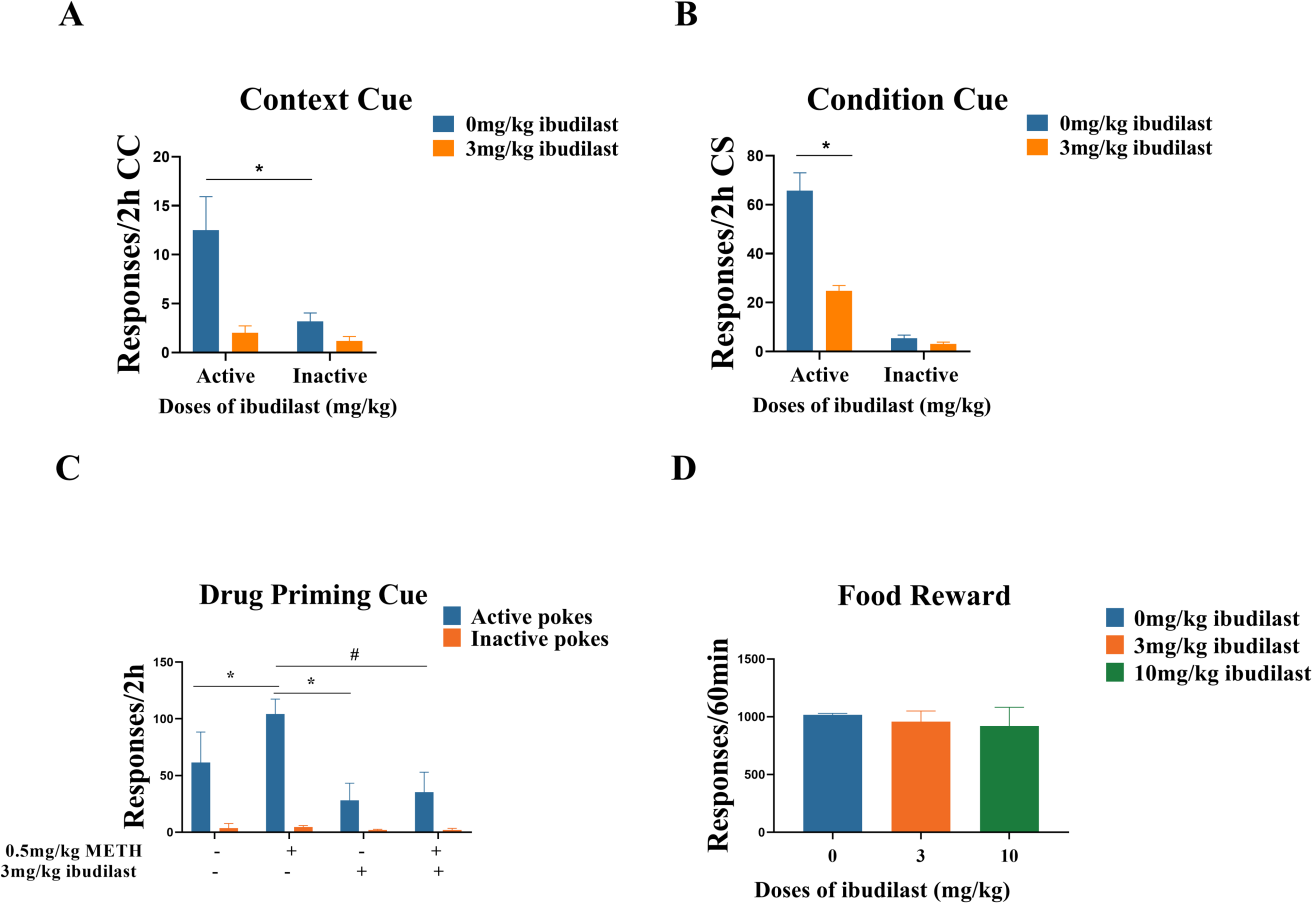
Effects of Ibudilast on drug‐seeking behaviour induced by cues or METH priming and food reward. **(A)** Effect of 3 mg/kg Ibudilast on context cue‐induced drug‐seeking behaviour (2‐h session). **(B)** Effect of 3 mg/kg Ibudilast on conditioned cue‐induced drug‐seeking behaviour (2‐h session). **(C)** Effect of 3 mg/kg Ibudilast on methamphetamine priming‐induced drug‐seeking behaviour (2‐h session). **(D)** Effects of 3 mg/kg and 10 mg/kg Ibudilast on oral sucrose self‐administration. Data are expressed as mean ± SD (*n* = 6). *p < 0.05, Ibudilast‐treated groups vs. Vehicle. #*p* < 0.05, 0.5 mg/kg METH + 3 mg/kg Ibudilast vs. 0.5 mg/kg METH.

We evaluated the effects of 3 mg/kg Ibudilast on context induced seeking behaviour after a 14‐day withdrawal period. As shown in Figure 2A, the statistics analysis indicated a significant impact of 3 mg/kg Ibudilast on active nose pokes (F _(1, 10)_ = 15.39, *p* = 0.025), while there were no significant differences in inactive nose pokes across the groups (F _(1, 10)_ = 1.779, *p* = 0.250). This suggests that 3 mg/kg Ibudilast inhibits drug‐seeking behaviour triggered by contextual cues. For another rat group, we tested reinstatement of METH‐seeking behaviour with cue‐induced reinstatement. As shown in Figure 2B, statistics analysis revealed a significant effect on active nose pokes (F _(1, 10)_ = 12.36, *p* = 0.0003), but no effect on inactive nose pokes (F _(1, 10)_ = 2.867, *p* = 0.177). Compared to Vehicle group, 3 mg/kg Ibudilast reduced the number of active nose pokes (*p* = 0.004), suggesting that 3 mg/kg Ibudilast could inhibit cue‐induced reinstatement.

We assessed METH‐primed seeking behaviour in METH‐trained animals divided into four groups (six animals per group): Vehicle group, METH‐primed group, 3 mg/kg Ibudilast group, and METH combined with 3 mg/kg Ibudilast treatment group. As shown in Figure 2C, a one‐way ANOVA (F _(3, 20)_ = 29.41, *p* < 0.0001) revealed a significant increase in active nose pokes compared to Vehicle group when METH was primed (*p* = 0.0007), indicating successful drug‐primed reinstatement. When METH priming and 3 mg/kg Ibudilast were administered simultaneously, 3 mg/kg Ibudilast suppressed drug‐primed reinstatement behaviour compared to the METH‐primed group (*p* < 0.0001).

To investigate the specificity of Ibudilast's inhibitory effects on the behaviour of METH‐addicted rats, we examined its impact on natural food reward behaviour. As shown in Figure 2D, there was no significant difference of sucrose pellets among the groups (F _(2, 15)_ = 5.29, *p* = 0.259), either 3 or 10 mg/kg Ibudilast had no significant effect on natural reward behaviour (*p* = 0.144, *p* = 0.171).

### 3.3 Effects of Ibudilast on Inflammation in PFC After METH Self‐Administration

**FIGURE 3:**
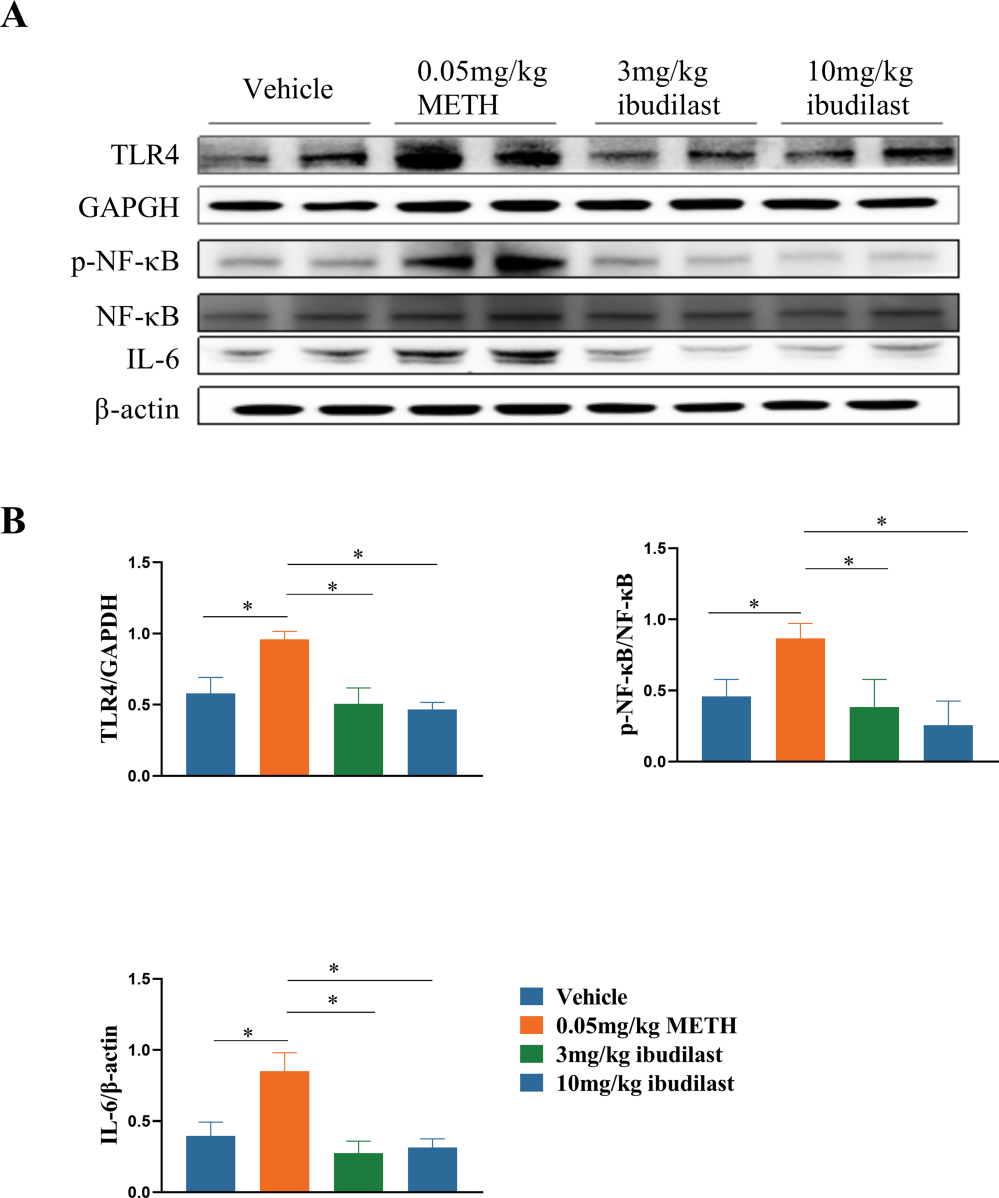
Effects of Ibudilast on inflammation in the prefrontal cortex after METH self‐administration. **(A)** Expression levels of TLR4, p‐NF‐κB and IL‐6 in the prefrontal cortex following treatment with 3 mg/kg and 10 mg/kg Ibudilast. **(B)** Quantitative analysis of TLR4, p‐NF‐κB and IL‐6 protein expression. *p < 0.05, 3 or 10 mg/kg Ibudilast‐treated groups vs. Vehicle or 0.05 mg/kg METH.

To further observe the relationship between Ibudilast and neuroinflammation induced by METH self‐administration, the relative levels of TLR4, p‐NF‐κB (phosphorylation NF‐κB) and IL‐6 in the PFC of each group were assessed. One‐way ANOVA statistics revealed a significant difference in the levels of TLR4 (Figure 3A,B, *F* = 19.85, *p* = 0.002), p‐NF‐κB (*F* = 12.22, *p* = 0.001) and IL‐6 (*F* = 23.04, *p* = 0.001) among the four groups. The multiple comparisons revealed TLR4 expression significantly increased in the PFC after 14 days (*p* = 0.043) compared to the Vehicle group. Treatment with 3 or 10 mg/kg Ibudilast inhibited expression of TLR4, and the expression of TLR4 in the PFC was significantly reduced compared to the 14‐day METH training group (*p* = 0.027, *p* = 0.001). p‐NF‐κB expression showed a similar to that of TLR4. The multiple comparisons revealed p‐NF‐κB expression significantly increased in the PFC after 14 days (*p* = 0.009) compared to the Vehicle group. Treatment with 3 or 10 mg/kg Ibudilast inhibited expression of TLR4, and the expression of TLR4 in the PFC was significantly reduced compared to the 14‐day METH training group (*p* = 0.030, *p* = 0.006). The multiple comparisons revealed IL‐6 expression significantly increased in the PFC after 14 days (*p* = 0.0318) compared to the Vehicle group. Treatment with 3 or 10 mg/kg Ibudilast inhibited expression of TLR4, and the expression of TLR4 in the PFC was significantly reduced compared to the 14‐day METH training group (*p* = 0.015, *p* = 0.024).

### 3.4 High Expression of TLR4 in Neurons and the Interaction Between Neurons and Microglia After METH Self‐Administration

**FIGURE 4:**
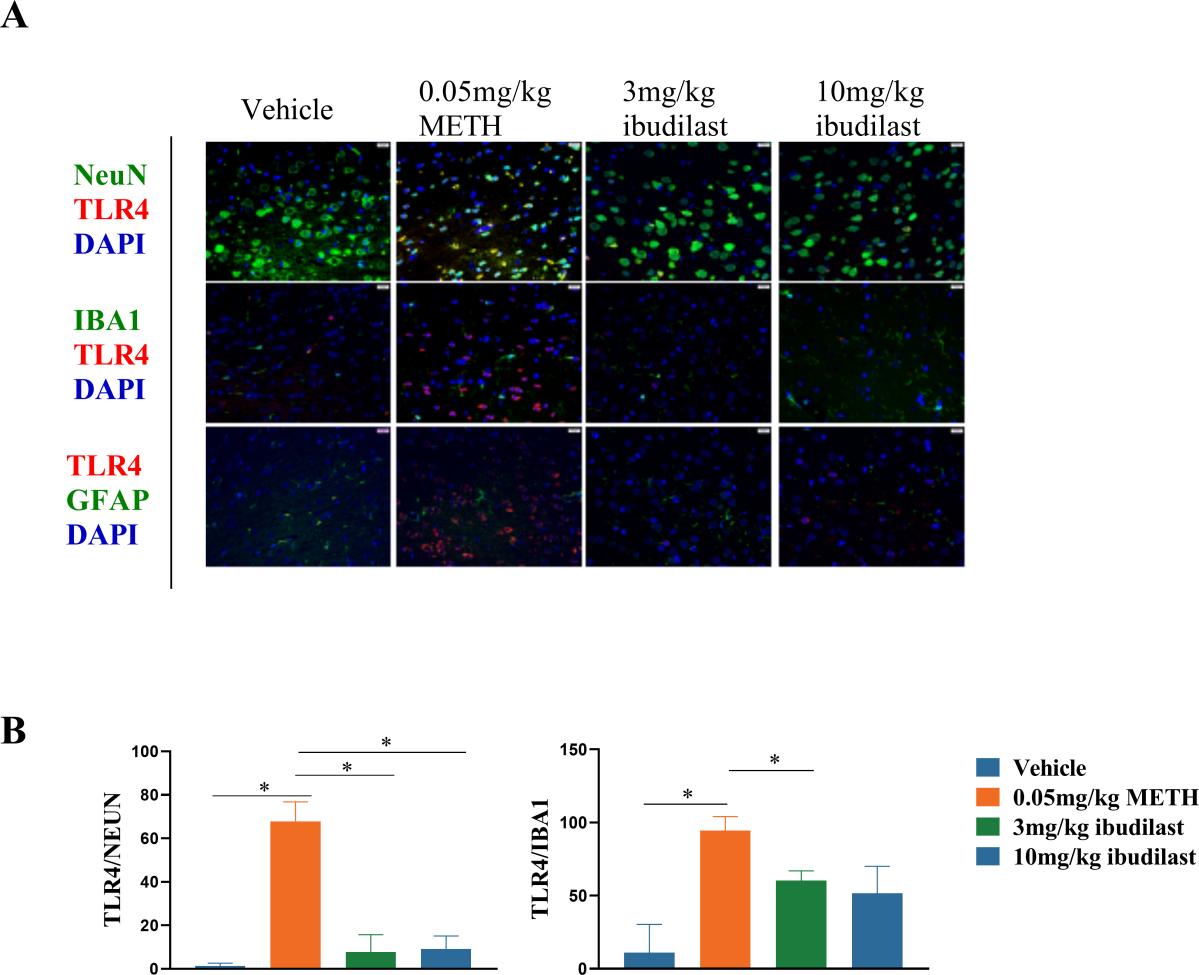
Effects of Ibudilast on neurons in the prefrontal cortex after METH self‐administration. **(A)** Expression of TLR4 in neurons, microglia and astrocytes following treatment with 3 and 10 mg/kg Ibudilast. **(B)** Quantitative analysis of TLR4 expression in neurons, microglia and astrocytes. *p < 0.05, 3 or 10 mg/kg Ibudilast‐treated groups vs. Vehicle or 0.05 mg/kg METH.

To determine the cellular localization of TLR4 expression, we performed immunofluorescence staining on coronal sections of the PFC (IL region) from Vehicle and METH addiction rats. As shown in Figure 4A,B, We used NeuN as a neuronal marker, IBA1 as a marker for microglia, and GFAP as a marker for astrocytes, co‐staining with TLR4. Compared to the Vehicle group, TLR4 expression was significantly elevated in neurons and microglia in the PFC of METH‐addicted rats, particularly in neurons. After administration of 3 or 10 mg/kg of Ibudilast, TLR4 expression was notably reduced. In contrast, TLR4 expressions in astrocytes remained low in both groups.

**FIGURE 5:**
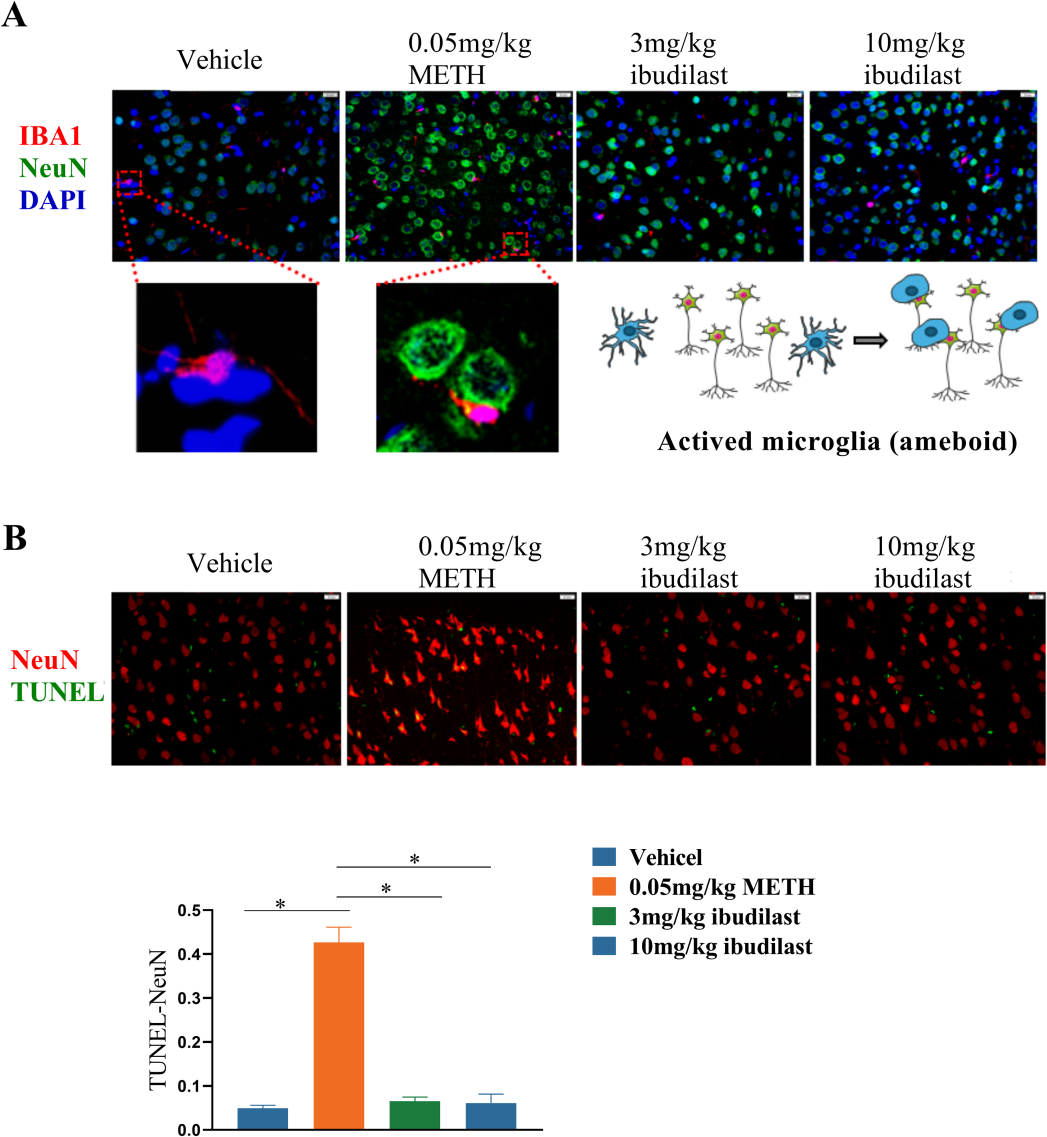
Effects of Ibudilast on neuron‐microglia crosstalk in the prefrontal cortex after METH self‐administration. **(A)** Analysis of neuron–microglia crosstalk following treatment with 3 mg/kg and 10 mg/kg Ibudilast. **(B)** Effect of 3 mg/kg and 10 mg/kg Ibudilast on TUNEL expression in neurons. *p < 0.05, 3 mg/kg or 10 mg/kg Ibudilast‐treated groups vs. Vehicle or 0.05 mg/kg METH.

In the METH addiction group, there was a significant visual overlap between IBA1 and NeuN immunoreactivity (Figure 5A), which was rarely observed in the Vehicle and Ibudilast‐treated groups. Imaging revealed a close spatial relationship between microglia and neurons, indicating that physical interactions between microglial and neuronal cells occur after METH addiction and are reversed by Ibudilast treatment. Additionally, TUNEL assay results showed that neurons in the METH addiction group exhibited high TUNEL expression (Figure 5B), which was reversed by Ibudilast treatment. In conclusion, our results suggest that neurons with high TLR4 expression undergo apoptosis and interact with microglia following METH addiction, and these interactions are reversed by Ibudilast treatment.

### 3.5 Microinjection of Ibudilast Into the PFC Reduced METH Infusions and Motivation

**FIGURE 6:**
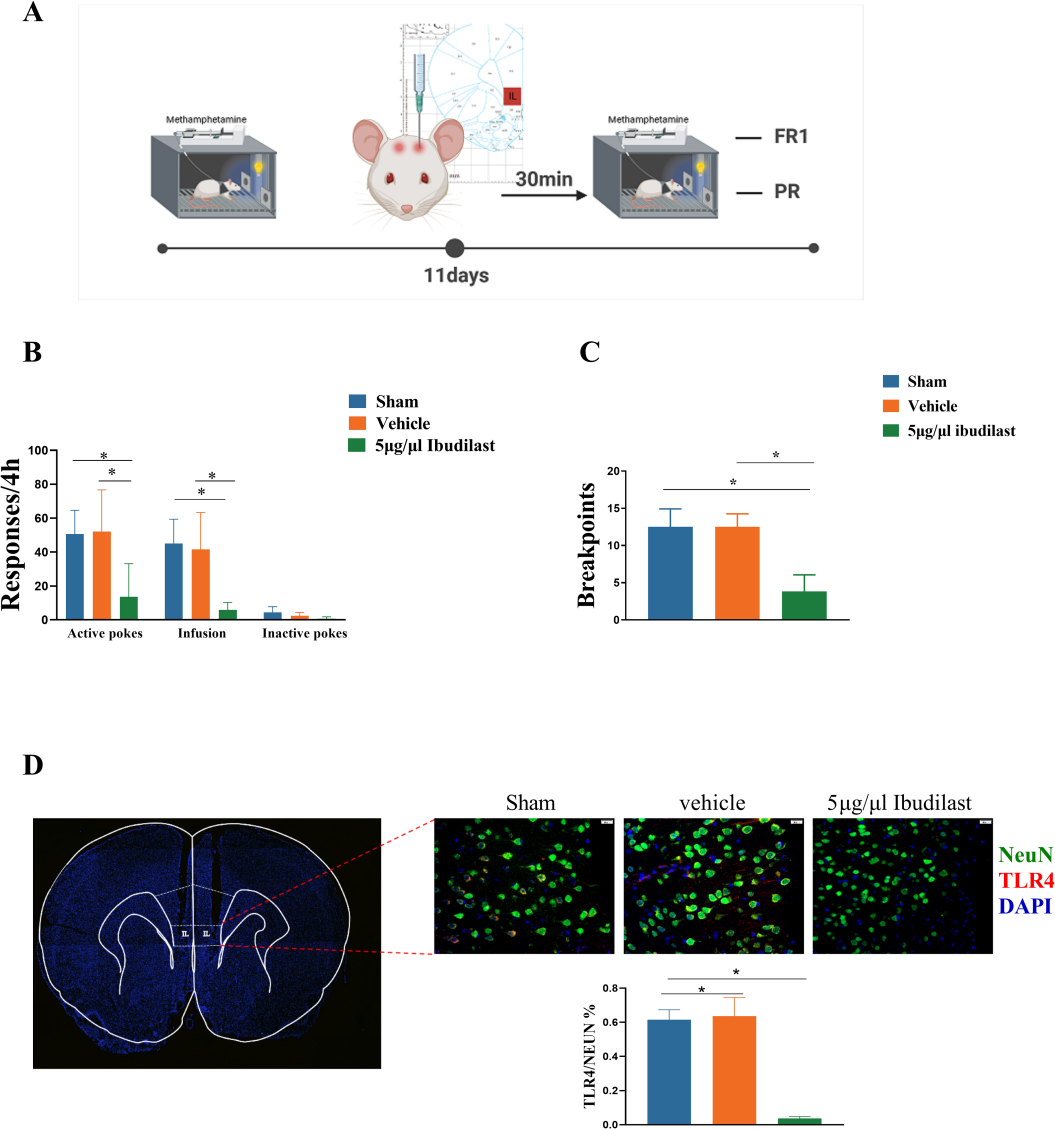
Effects of microinjection of Ibudilast on METH self‐administration and motivation. **(A)** Schematic representation of the experimental design and procedure. **(B)** Effects of microinjection of Ibudilast on METH self‐administration. **(C)** Effects of microinjection of Ibudilast on breakpoints.**(D)** Quantitative analysis of TLR4 expression in neurons after microinjection of Ibudilast. *p < 0.05, microinjection of 5 μg/μL Ibudilast groups vs. sham or Vehicle.

As shown in Figure 6A, we performed local injections of Ibudilast into the PFC of rats in a METH addiction model. The doses of Ibudilast and the pretreatment times were based on previous studies [26]. Ibudilast pretreatment significantly inhibited the number of active nose pokes (F _(2, 15)_ = 8.466, *p* = 0.003, Figure 6B) and METH infusions (F _(2, 15)_ = 14.08, *p* = 0.001) in a dose‐dependent manner, but had no significant effect on inactive nose pokes (F _(2, 15)_ = 1.489, *p* = 0.299). Figure 6B shows that Post‐hoc Tukey's tests showed that, compared to sham, Ibudilast significantly reduced the number of active nose pokes (*p* = 0.0049)and compared to Vehicle pretreatment, Ibudilast significantly reduced the number of active nose pokes (*p* = 0.019), and compare to sham of METH infusions, Ibudilast significantly reduced the number of infusions (*p* = 0.0005), compare to Vehicle of METH infusions, Ibudilast significantly reduced the number of infusions (*p* = 0.011).

Next, we examined whether Ibudilast could affect the motivation to self‐administer METH using a PR schedule. In this experiment, rats had to perform progressively more active nose pokes to receive the corresponding reward (METH infusion) until they reached a breakpoint. ANOVA revealed that Ibudilast dose‐dependently decreased the METH breakpoint F _(2, 15)_ = 32.27, *p* < 0.0001; (Figure 6C). Post‐hoc Tukey's tests showed that, compared to saline pretreatment, Ibudilast pretreatment significantly reduced the breakpoint (*p* = 0.0002).

Finally, we assessed the effect of Ibudilast on TLR4 expression in neurons within the PFC using three treatment groups after METH self‐administration:Sham, Vehicle, and 0.5 μg/L Ibudilast. One‐way ANOVA revealed a significant effect of Ibudilast treatment on TLR4 expression in neurons (F _(2, 6)_ = 68.47, *p* < 0.0001; Figure 6D). Post‐hoc comparisons indicated that Ibudilast significantly reduced TLR4 expression in neurons compared to both the Vehicle (*p* < 0.0001) and sham groups. No significant difference was observed between the sham and Vehicle groups (*p* = 0.9238).

## 4 Discussion

The pathological features of METH addiction are multifaceted, including neuronal damage, neuroinflammation, dysfunction of the reward system, and changes in brain structure [27]. These pathological changes are intertwined and collectively contribute to the persistence of addictive behaviours as well as cognitive and emotional impairments. Ibudilast, as a TLR4 inhibitor and glial cell modulator, has been hypothesized to possess neuroprotective properties. Previous studies have reported that Ibudilast effectively alleviates METH‐induced microglial activation and reduces neuroinflammatory responses [28]. However, prior to this study, the specific molecular mechanisms by which Ibudilast affects neurons and microglial cells in the PFC of the METH self‐administration rat model remained unclear.

METH, a potent central nervous system stimulant, induces a strong rewarding effect by increasing dopamine release and inhibiting its reuptake, thereby promoting addictive behaviours [29]. In the present study, we used a self‐administration model to assess the effects of Ibudilast on METH self‐administration behaviour and motivation, as well as its impact on relapse and natural reward behaviour. The results demonstrated that Ibudilast significantly reduced the effective nose‐poke frequency and total intake of METH, as well as relapse behaviour induced by environmental and conditioned cues, indicating that Ibudilast can inhibit METH‐induced self‐administration behaviours. However, Ibudilast did not significantly affect natural reward behaviour, suggesting its specificity in METH addiction.

The PFC plays a crucial role in executive functions, decision‐making and self‐control, and is the primary area affected by cognitive impairments following METH use [30]. TLR4 (Toll‐like receptor 4) is a pattern recognition receptor involved in immune responses and has been shown to play a central role in neuroinflammation [3].Studies indicate that TLR4 activation can trigger downstream inflammatory factors via the NF‐κB signalling pathway, initiating inflammatory responses in neural cells. In METH‐induced neuroinflammation, the upregulation of TLR4 is considered one of the key factors in the excessive activation of microglia and neurons [28]. Our results show that in the METH self‐administration model, TLR4, p‐NF‐κB and IL‐6 levels were elevated in the prefrontal cortex. Ibudilast significantly reduced these markers, suggesting that Ibudilast can mitigate the METH‐induced inflammatory response in the PFC by downregulating the TLR4‐p‐NF‐κB‐IL‐6 pathway.

METH use typically leads to excessive activation of microglia, which release pro‐inflammatory cytokines such as IL‐1β, TNF‐α, and IL‐6, exacerbating neuronal damage [31]. However, the direct mechanisms by which METH induces neuronal damage and excessive activation remain unclear. In our study, we found that neurons in the PFC showed high expression of TLR4 and an increase in apoptotic markers (TUNEL). After Ibudilast treatment, the number of TUNEL‐positive cells was significantly reduced, indicating a protective effect of Ibudilast against neuronal apoptosis. This effect may be mediated through Ibudilast's inhibition of the TLR4/NF‐κB signalling pathway and the release of downstream inflammatory factors, alleviating the neuroinflammatory response and reducing neuronal apoptosis. Additionally, Ibudilast, as an anti‐inflammatory agent, may indirectly prevent apoptosis by reducing microglial overactivation. In the METH self‐administration condition, microglia in the PFC showed significant activation, characterized by microglial proliferation and amoeboid morphology. After Ibudilast treatment, this microglial activation was effectively suppressed. This finding is consistent with previous studies, which have shown that Ibudilast can inhibit excessive microglial activation and reduce neuroinflammatory responses [24]. Ibudilast inhibits the activation of the NF‐κB and MAPK signalling pathways, thereby preventing the production of pro‐inflammatory cytokines in microglia and mitigating METH‐induced neurotoxicity. Importantly, the interaction between microglia and neurons via TLR4 signalling may create a feedback loop, where microglial activation exacerbates neuronal damage, and neuronal damage further activates glial cells. By inhibiting TLR4 activation in both neurons and microglia, Ibudilast may disrupt this cycle, reducing neuroinflammation and alleviating neuronal apoptosis. The interaction between glial cells and neurons in neuroinflammation, and their contribution to METH‐induced neurotoxicity, underscores the importance of therapeutic interventions targeting both cell types simultaneously.

Neurons and microglia in the cerebral cortex interact in specific spatial structures and functional relationships. Under normal conditions, neurons and microglia maintain a certain spatial distance and coordinate through synaptic and immune responses to perform critical functions such as neurotransmission and immune surveillance [32]. However, in various pathological conditions, particularly those induced by drug addiction, neuroinflammation or oxidative stress, the spatial relationship between neurons and microglia can become disordered or reorganized [33]. Such spatial folding is usually accompanied by excessive activation of microglia, release of inflammatory factors and neuronal damage or apoptosis [34]. In our study, we observed a distinct folding phenomenon in the spatial relationship between neurons and microglia in the METH‐treated group, which may reflect microglial activation and neuronal network remodelling induced by neuroinflammation. Specifically, METH‐induced neuroinflammation overactivated microglia, causing them to migrate and alter their relative position to neurons, thereby exacerbating neuronal damage. Following Ibudilast treatment, the abnormal spatial folding or rearrangement of neurons and microglia was effectively inhibited, indicating that Ibudilast can influence the interaction between neurons and microglia.

Finally, the specific administration of Ibudilast to the PFC resulted in reduced METH self‐administration behaviour and motivation, further supporting the specific role of the TLR4‐p‐NF‐κB pathway in METH self‐administration behaviour. Meanwhile, the immunostaining showed that local injection of Ibudilast into the PFC significantly reversed the increased TLR4 expression in neurons in the PFC after METH self‐administration. This restoration of neuronal TLR4 expression may represent a key mechanism by which Ibudilast modulates the neuroimmune environment and reduces neuronal damage, supporting its potential neuroprotective role in reducing METH‐induced neuroinflammation and neuronal apoptosis.

In summary, our findings suggest that Ibudilast alleviates METH self‐administration behaviour by inhibiting the excessive activation of neurons and microglia in the PFC through the TLR4‐p‐NF‐κB pathway, reducing neuronal apoptosis and improving the interaction between neurons and microglia. These results highlight Ibudilast's potential neuroprotective role in METH‐induced neurotoxicity.

## 5 Conclusion

This study demonstrates that Ibudilast effectively mitigates METH‐induced neurotoxicity by targeting the prefrontal cortex, a key brain region implicated in METH addiction and associated cognitive impairments. Ibudilast significantly reduced METH self‐administration behaviour, relapse, and addiction motivation, while sparing natural reward behaviours, highlighting its specificity for METH addiction.

Mechanistically, Ibudilast alleviated neuroinflammation by downregulating the TLR4‐p‐NF‐κB‐IL‐6 signalling pathway, suppressing the overactivation of neurons and microglia, and inhibiting the release of pro‐inflammatory cytokines. Furthermore, Ibudilast reduced the number of TUNEL‐positive cells, thereby protecting neurons from apoptosis and improving pathological interactions between neurons and microglia. These effects preserved neuronal integrity and alleviated neuroinflammatory damage.

These findings underscore the neuroprotective role of Ibudilast in the METH self‐administration model and demonstrate its potential as a therapeutic agent for treating METH addiction. Future studies should further explore the specific molecular mechanisms underlying Ibudilast's modulation of neuron‐microglia interactions and its clinical application potential in the field of addiction medicine.

## Author Contributions

Conceptualization: F.W. and W.Z. Methodology: F.W., H.L., L.H., S.C., D.Z., Y.Z., M.W., Y.W., M.L. Data analysis: F.W., H.L., Y.K. Original draft preparation: F.W. Review and editing: W.Z., Y.K., H.L. Supervision: W.Z. Funding acquisition: F.W. and W.Z. W.Z. is the guarantor of this work and, as such, has full access to all the data in the study and takes responsibility for the integrity of the data and the accuracy of the data analysis.

## Conflicts of Interest

The authors declare no conflicts of interest.

## Data Availability

The data that support the findings of this study are available from the corresponding author upon reasonable request.
